# Do Alterations in the Rate of Gastric Emptying after Injection Sclerotherapy for Oesophageal Varices Play any Role in the Development of Portal Hypertensive Gastropathy?

**DOI:** 10.1155/1999/27037

**Published:** 1999-04

**Authors:** K. K. Balan, J. S. Grime, R. Sutton, M. Critchley, S. A. Jenkins

**Affiliations:** 1 Department of Nuclear Medicine Royal Liverpool University Hospital Liverpool United Kingdom; 2 Department of Surgery Royal Liverpool University Hospital Liverpool United Kingdom; 3 Academic Department of Surgery Postgraduate Medical School Morriston Hospital Swansea United Kingdom; 4 Department of Nuclear Medicine Box. No. 170 Addenbrooke's Hospital Cambridge CB22QQ United Kingdom

## Abstract

Bleeding from portal hypertensive gastropathy (PHG) has been estimated to account for upto 30% of all upper gastrointestinal haemorrhage in patients with cirrhosis and portal hypertension. Although portal hypertension seems to be an essential prerequisite, the precise mechanisms responsible for the development of PHG are unknown. The aim of this study was to examine the role of injection sclerotherapy of oesophageal varices in the development of PHG. Gastric emptying was studied using a radionuclide test meal with the emptying characteristics of a slow liquid in 57 patients with cirrhosis and/or portal hypertension (median age 53 yrs), of whom 34 had received injection sclerotherapy for their oesophageal varices and 20 normal healthy volunteers (median age 42 yrs). As vagal damage is associated with more rapid emptying of liquids, despite hold up of solids, this technique might be expected to demonstrate such damage if gastric emptying was accelerated. The results indicated that there was no difference in the rate of gastric emptying between normal healthy volunteers and portal hypertensive patients. However, patients who had received injection sclerotherapy emptied their stomachs faster than those who had not (*p*<0.05). Furthermore, the speed of gastric emptying correlated directly with the number of injections (*r*=0.41; *p*=0.02) and the volume of sclerosant injected (*r*=0.39; *p*=0.03). These observations suggest that injection sclerotherapy for oesophageal varices results in disturbances of gastric emptying that may contribute to the pathogenesis of portal hypertensive gastropathy.


*HPB Surgery*, 1999, Vol. 11, pp. 141–150
Reprints available directly from the publisher
Photocopying permitted by license only

© 1999 OPA (Overseas Publishers Association) N.V.
Published by license under
the Harwood Academic Publishers imprint,
part of The Gordon and Breach Publishing Group.
Printed in Malaysia.

### Do Alterations in the Rate of Gastric Emptying after Injection Sclerotherapy for Oesophageal Varices Play any Role in the Development of Portal Hypertensive Gastropathy?

K. K. BALAN[a,*], J. S. GRIME[a], R. SUTTON[b], M. CRITCHLEY[a] and S. A. JENKINS[c]

[a] *Departments of Nuclear Medicine, Royal Liverpool University Hospital, Liverpool;*
[b] *Department of Surgery, Royal Liverpool University Hospital, Liverpool;*
[c] *Academic Department of Surgery, Postgraduate Medical School, Morriston Hospital, Swansea, UK*

*(Received 30 November 1995; In final form 30 April 1998)*

**Bleeding from portal hypertensive gastropathy (PHG) has been estimated to account for upto 30% of all upper gastrointestinal haemorrhage in patients with cirrhosis and portal hypertension. Although portal hypertension seems to be an essential prerequisite, the precise mechanisms responsible for the development of PHG are unknown. The aim of this study was to examine the role of injection sclerotherapy of oesophageal varices in the development of PHG. Gastric emptying was studied using a radionuclide test meal with the emptying characteristics of a slow liquid in 57 patients with cirrhosis and/or portal hypertension (median age 53 yrs), of whom 34 had received injection sclerotherapy for their oesophageal varices and 20 normal healthy volunteers (median age 42 yrs). As vagal damage is associated with more rapid emptying of liquids, despite hold up of solids, this technique might be expected to demonstrate such damage if gastric emptying was accelerated. The results indicated that there was no difference in the rate of gastric emptying between normal healthy volunteers and portal hypertensive patients. However, patients who had received injection sclerotherapy emptied their stomachs faster than those who had not ($p < 0.05$). Furthermore, the speed of gastric emptying correlated directly with the number of injections ($r = 0.41$; $p = 0.02$) and the volume of sclerosant injected ($r = 0.39$; $p = 0.03$). These observations suggest that injection sclerotherapy for oesophageal varices results in disturbances of gastric emptying that may contribute to the pathogenesis of portal hypertensive gastropathy.**

*Keywords:* Portal hypertensive gastropathy, gastric emptying, injection sclerotherapy, oesophageal varices

### INTRODUCTION

Bleeding from the portal hypertensive gastric mucosa has been estimated to account for up to 30% of upper gastrointestinal haemorrhage in patients with cirrhosis and portal hypertension [1–5]. Although variously described as "haemorrhagic gastritis" [6], "acute gastric erosions"

*Address for correspondence: Department of Nuclear Medicine, Box. No. 170, Addenbrooke's Hospital, Cambridge, CB2 2QQ.

[7], "acute mucosal lesions" [8], "alcoholic gastritis" *etc.* [29] the term portal hypertensive gastropathy (PHG) is currently accepted to describe the gastric mucosal lesions responsible for this condition [10]. The precise mechanisms responsible for PHG are incompletely understood but portal hypertension is considered to be an essential prerequisite [11 – 13]. Injection sclerotherapy has been reported to exacerbate portal hypertensive gastropathy, [5 – 14, 15], although the mechanisms involved are unknown. One possible explanation for the deleterious effect of injection sclerotherapy on PHG is the gastric mucosal congestion which results from the obliteration of lower oesophageal veins. However, this is likely to arise only if blood flow in the varices is cephalad which is not always the case [16, 17]. A further possibility is that extravasation of sclerosant into the mediastinum can result in vagal damage producing a delay in the rate of gastric emptying. Such a delay in gastric emptying may prolong the exposure of already susceptible gastric mucosa of portal hypertensive patients to the deleterious effects of acid and pepsin and exacerbate PHG. Since this possibility has not been hitherto examined, the aim of the present study was to investigate gastric emptying in patients who had received varying courses of elective sclerotherapy and differing amounts of sclerosant to obliterate their varices.

### SUBJECTS AND METHODS

### Subjects

The study was performed in 57 patients (33 males and 24 females, median age 53 years), of whom 50 had cirrhosis of the liver (alcoholic 32, primary biliary cirrhosis 7, cryptogenic 5, others 6) and 7 with extrahepatic portal hypertension and 20 normal healthy volunteers (male 14, female 6, median age 42 years). Clinical details on each patient, including the number of previous sclerotherapies and the total volume of sclerosant (ethanolamine oleate) injected, were obtained from the hospital case notes. All patients has undergone extensive hepatic investigations prior to the study, to establish the aetiology of the liver disease, and the extent of hepatic dysfunction. The aetiology of cirrhosis was confirmed by liver biopsy in all patients. Patients with extra-hepatic pathology had overt evidence of portal hypertension including oesophageal varices and or/ascites. None of the patients had active peptic ulceration at the time of study. Patients with severe cardiac, renal, and pulmonary disease were excluded from the study. Also excluded from the study were those patients admitted for an acute variceal bleed, severe ascites or severe clotting disorders and non-variceal upper gastrointestinal bleeding. No patient had undergone any gastric surgery. Ethical approval was obtained from the Hospital Ethical Committee. All the studies were conducted after obtaining written informed consent from all patients and volunteers, and ARSAC approval to carry out radionuclide gastric emptying studies.

### Methods

#### *Gastric Emptying Studies*

All gastric emptying studies were carried out at least one week following sclerotherapy and after a 12 h fast. Drugs that could influence gastric motility were discontinued 48 h prior to the study. The rate of gastric emptying (GE) was measured using a semi-solid meal [18] which consisted of 10 MBq of $^{99}Tc^{m}$-labelled bran incorporated into porridge made from Ready-Brek® and milk, two cheese sandwiches and a cup of tea. The total volume of the meal was 510 ml, the weight 430 g and the calorific content 2.7 MJ. Each patient sat at 45° on a chair in front of a large field of view gamma camera interfaced to a digital computer, and acquisition of the data was commenced at the beginning of the meal at a rate one frame per minute. At the end of the study, a composite image of the stomach was obtained by summing the first ten frames of the study. Two regions of interest (ROIs) were then drawn on this image, defining the stomach content and a background area. The counts in each ROI on each frame of the study were then obtained and a gastric emptying curve and background curve produced. A background corrected gastric emptying curve was derived by subtracting the individual background curve value multiplied by the quotient of the number of the cells in the gastric ROI and the number of the cells in the background ROI from each value of the gastric curve at each time interval. After correcting for radioactive decay the gastric empltying curve was finally displayed as a percentage emptying curve, by dividing each point of the curve by the maximum curve value and then multiplying by 100. The time taken for 50% GE ($t\,1/2$) was used to express the results.

### STATISTICAL ANALYSIS

The area under the curve method was used for analysis of GE. Differences between groups were assessed by Mann-Whitney U or Kruskal-Wallis H tests. The Spearman's rank correlation coefficient was used to determine the association between two variables. A '*p*' value of less than 0.05 was considered to be statistically significant.

### RESULTS

Of the 57 patients studies, 44 had oesophageal varices and 34 had received previous injection sclerotherapy. The oesophageal varices had been completely obliterated in 17 of these patients for periods varying from 5 months to 7 years. The number of sclerotherapies ranged from 1–17 (median 5) and the volume of sclerosant injected from 5.5–86.5 ml (median 39 ml).

As shown in Figure 1, there was no significant difference in the rate of GE between normal

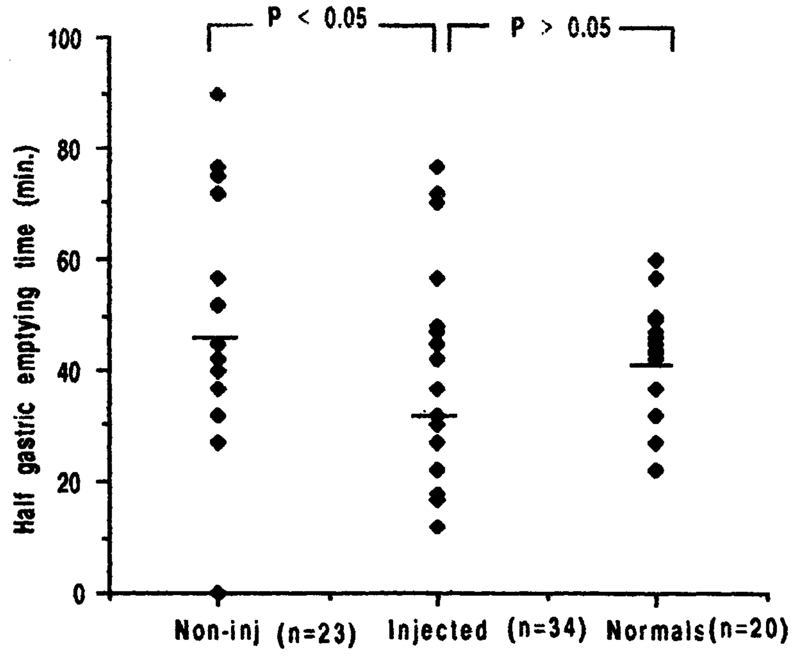

FIGURE 1 Gastric emptying ($t\,1/2$) of patients who had not received injection sclerotherpy (Non-inj), patients who had received elective injection sclerotherapy (Injected) and normal healthy volunteers (Normals).

subjects (median 57 min., range 30–67 min.) and patients who had not received injection sclerotherapy (median 56 min., range 39–77 min.). However, the rate of GE was significantly faster in patients who had received previous injection sclerotherapy for their oesophageal varices compared to cirrhotic patients without oesophageal varices and those with varices who had received no sclerotherapy (median 46 min., range 23–76 min.). There was no difference in the rate of GE between 17 patients with completely obliterated varices (median 56 min., range 39–77 min.) and 17 others who had received injection sclerotherapy but whose varices were still patent (median 58 min., range 40–77 min.). However, the rate of GE correlated significantly with the number ($r = -0.41$; $p = < 0.02$) of previous sclerotherapy sessions (Fig. 2) and the volume ($r = -0.39$; $p = < 0.03$) of sclerosant injected (Fig. 3).

### DISCUSSION

It has been suggested that injection sclerotherapy may play a role in the gastric mucosal changes characteristic of PHG. Thus, in a small uncontrolled series, McCormack *et al.* [5] observed that PHG appeared for the first time after successful obliteration of oesophageal varices by sclerotherapy in 6 patients. However in another 5 patients, variceal eradication appeared to coincide with disappearance of gastric lesions. Sarin *et al.* [14] reported a high incidence of a "mosaic like" pattern in the gastric mucosa of patients following obliteration of their varices. However, in that study, the incidence of PHG prior to sclerotherapy was unknown, making it impossible to ascertain the role of sclerosis in the pathogenesis of the condition. D'Amico *et al.* [15] observed that PHG tended to appear and never to improve during a course of elective sclero-

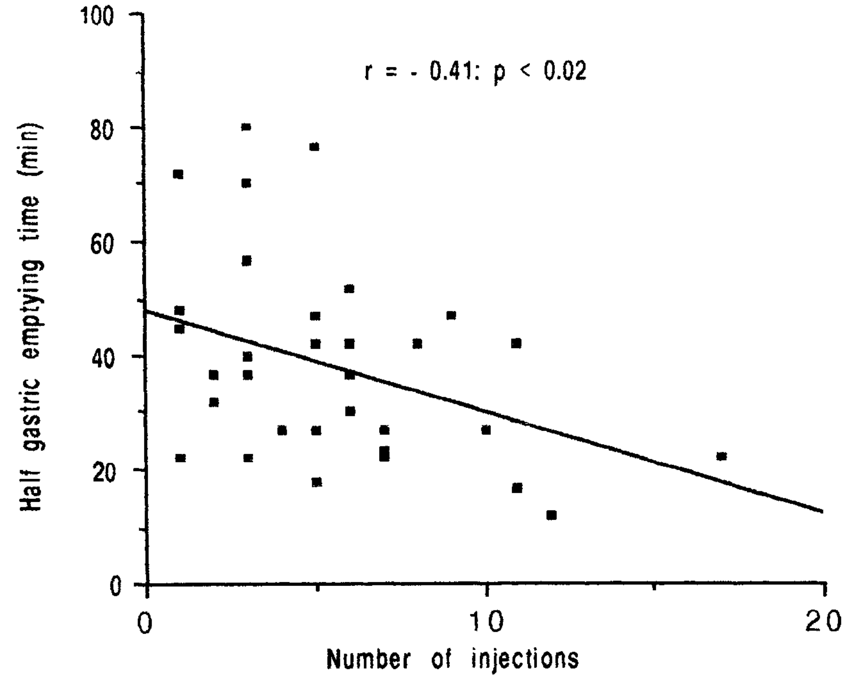

FIGURE 2 Correlation between gastric emptying ($t\,1/2$) and the number of injection sclerotherapy sessions in patients with esophageal varices.

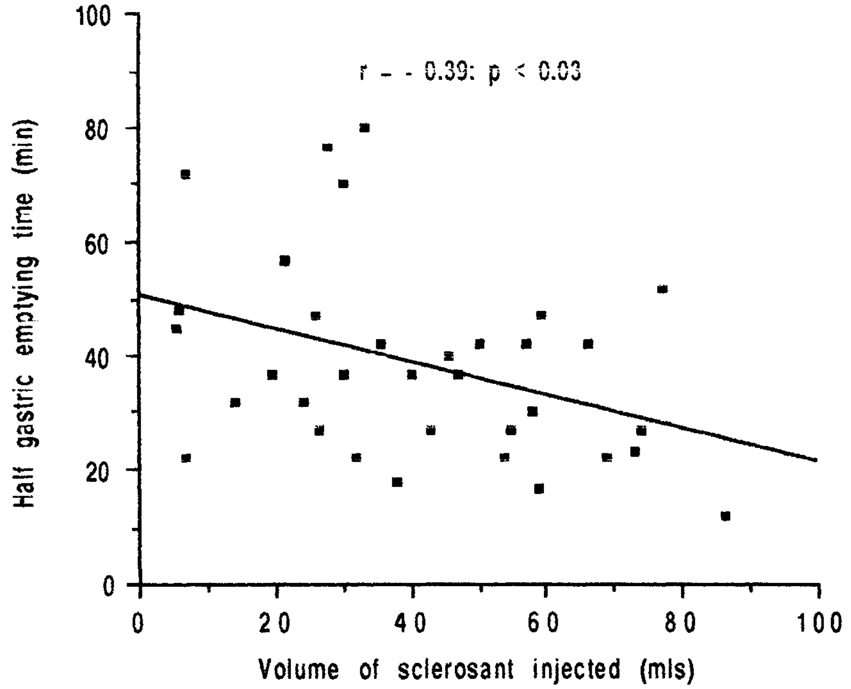

FIGURE 3 Correlation between gastric emptying ($t\,1/2$) and the volume of scerosant injected in patients with esophageal varices.

therapy, and in some patients occurred before obliteration of the varices was achieved. Furthermore, these investigators also noted that the number of overt haemorrhages from PHG was remarkably increased after sclerotherapy. Kotzampassi *et al.* [19] reported that a highly statistically significant number of patients developed PHG within 6 months after variceal obliteration by sclerotherapy. Thus 18 out of 20 of their patients with normal gastric mucosa prior to sclerotherapy developed moderate to severe PHG after obliteration of their varices. Furthermore 17 patients with mild gastropathy developed moderate to severe PHG after sclerotherapy.

Although the foregoing discussion indicates an increase in the incidence of PHG after injection sclerotherapy, the precise mechanism remains unclear. One possible explanation is that the gastric mucosal changes occur as a result of alterations in local haemodynamics following obliteration of the lower oesophageal veins. Previous studies [16, 17] have shown that the blood flow patterns in oesophageal varices may not always be cephalad but can often be towards the stomach offering an explanation for the observation that some patients develop PHG, whereas others do not.

Another possible explanation for the effects of injection sclerotherapy on the development of PHG is that extravasation of sclerosant into the mediastinum results in a "chemical vagotomy" which may be responsible for the alterations in oesophageal motility reported after multiple sessions of sclerotherapy [20, 21]. If this is indeed the case, then a "chemical vagotomy" should also delay gastric emptying of solids which may exacerbate PHG, by exposing the already susceptible gastric mucosa of portal hypertensive patients to the deleterious effects of acid and pepsin. There is some indirect evidence to support this hypothesis. Thus in portal hypertensive rats, restraint results in the development of gastric lesions and haemorrhage, which are abolished by truncal vagotomy and pyloroplasty [22]. Pyloroplasty was introduced in man to accelerate gastric emptying in patients who developed stasis after truncal vagotomy alone. Therefore a chemical vagotomy without any drainage procedure should promote gastric stasis and exacerbate PHG by exposing the gastric mucosa to the deleterious effects of gastric contents for longer periods.

The results of present study suggest that there is no difference in the rate of GE of a semi-solid meal between normal subjects and patients who had received injection sclerotherapy. These observations conflict with those of Bonvoisin *et al.* [23] who demonstrated a delay in GE of both solids and liquids, the latter more pronounced, in 9 patients with alcoholic cirrhosis who had undergone sclerotherapy, compared to 8 normal healthy controls. Stein *et al.* [24] has studied gastric motility in two patients before and after injection sclerotherapy. However, the number of subjects included in these two studies are too small to properly assess the significance of the results. Nevertheless, the conflicting findings between the previous study by Bonvoisin *et al.* [23] and our own cannot be explained by the difference in the test meal used in the two studies, since the former had demonstrated a delay in GE of both solid and liquid meals whereas we observed a faster rate of GE using a semi-solid meal. However, if vagal damage resulted in abnormalities of GE after injection sclerotherapy, solid meals should empty slower and liquids or semi-solids faster than in normal controls. Therefore, the observations of Bonvoisin *et al.* [23] cannot be explained in terms of vagal damage *per se*. In the present study, not only the rate of GE was significantly faster in patients who had received injection sclerotherapy compared to those who had not, although there was considerable overlap between the two groups. These observations would suggest that in patients who received a small number of sclerotherapy sessions and hence a small volume of sclerosant, there is minimal damage and therefore little or no damage in the rate of GE. However, as the number of courses of injection sclerotherapy and the volume of the sclerosant administered increases the rate of GE increases as the vagal damage becomes more pronounced. This suggestion is supported by the positive correlations between the rate of GE and both the number of injections as well as the volume of sclerosant injected. These observations would suggest that injection sclerotherapy plays an independent role in causing the faster GE of a semi-solid meal in portal hypertensive patients. The most likely explanation for this increased rate of GE after injection sclerotherapy is the effect of the procedure on vagal function. The proximity of the anterior and posterior trunks of vagus nerve to the lower oesophagus probably makes them vulnerable to the deleterious effects of any sclerosant that extravasates into the mediastinum. The test meal used in the present study has the emptying characteristics of a slow liquid. Therefore, it would appear that the effects of sclerotherapy on the rate of GE is analogous to that which occurs after highly selective vagotomy whereby liquids empty faster as a result of loss of receptive relaxation of the proximal stomach [21, 26]. Furthermore, gastric emptying of liquids has also been noted to be faster after truncal vagotomy [27] which affects gastric antral motility, resulting in a delay in emptying of solids. Thus the degree of damage to the vagus can theoretically dictate the nature of any resulting GE abnormality. A delay in gastric emptying can expose the already vulnerable gastric mucosa of portal hypertensive patients to the deleterious effects of endogenous "aggressive" substances like acid and pepsin and thereby cause aggravation of or the development of PHG.

Previous studies have demonstrated disturbance in autonomic nervous function in cirrhosis regardless of its aetiology [28, 29]. If this is indeed the case, gastric motor function can be affected in a way analogous to different vagotomies, depending on the degree of vagal denervation. Autonomic function, however, was not formally assessed in our study population. In a recent study by Isobe *et al.* [30] in cirrhotics, although there was evidence of impaired cardiac autonomic function, there was a poor correlation between this and gastric emptying. Thus, it would seem unlikely that a disturbance in autonomic function was responsible for the alterations in the rate of GE observed in the present study.

In summary, the results of this study suggest that disturbances of GE may occur after injection sclerotherapy particularly in those patients who have received a large number of courses of sclerosis and hence volume of sclerosant. Furthermore, these disturbance in GE may contribute to the development of PHG, since the patterns of abnormal GE observed after sclerotherapy particularly in those patients who had received a large number of sclerotherapies and hence a large volume of sclerosant appear to be consistent with vagal damage. However, there was considerable overlap between the rate of GE in patients who had or had not received injection sclerotherapy. Further larger studies are therefore required between patients who have undergone a larger number of courses of injection sclerotherapy and those who have received less or no sclerosis before the clinical significance of the findings in this study can be confirmed. Furthermore, data from $p^H$-metry and manometry may also be required for precise evaluation of "chemical vagotomy".

### *References*

[1] McCray, R. S., Martin, F., Amir-Ahmadi, F. and Shahan, D. G. (1969). Zamcheck. Erroneous diagnosis of haemorrhage from oesophageal varices. *Am. J. Dig. Dis.*, **14**, 755–761.

[2] Dagradi, A. E., Sanders, D. and Stempien, J. (1954). The Sources of upper gastrointestinal bleeding in liver cirrhosis. *Am. J. Gastroenterol.*, **54**, 852–855.

[3] Walram, S., Davis, M., Nunnerley, H. and Williams, R. (1974). Emergency endoscopy after gastrointestinal haemorrhage in 50 patients with portal hypertension. *Br. Med. J.*, **4**, 94–96.

[4] Franco, D., Durandy, Y., Departe and Bismuth, H. (1977). Upper gastrointestinal haemorrhage in hepatic cirrhosis: causes and relation to hepatic failure and stress. *Lancet.*, **1**, 218–220.

[5] McCormack, T. T., Sims, J., Eyre-Brook, I., Kennedy, H., Goepel, J. and Johnson, A. G. *et al.* (1985). Gastric lesions in portal hypertension: inflammatory gastritis or congestive gastropahty? *Gut*, **26**, 1226–1232.

[6] Mitchell, K. J., MacDougal, B. R. D., Silk, D. B. A. and Williams, R. (1982). A prospective reappraisal of emergency endoscopy in patients with portal hypertension. *Scand. J. Gastroent.*, **17**, 965–968.

[7] Lebrec, D., De Fleury, P., Rueff, B., Nahum, H. and Benhamou, J. P. (1980). Portal hypertension, size of oesophageal varices and the risk of gastrointestinal bleeding in alcholic cirrhosis. *Gastroenterology*, **79**, 1139–1344.

[8] Teres, J., Bordas, J. M., Bru, C., Diaz, F., Bruguera, M. and Rodes, J. (1976). Upper gastrointestinal bleeding in cirrhosis: clinical and endoscopic correlation. *Gut*, **17**, 37–40.

[9] Khodadoost, J. and Glass, G. B. J. (1972). Erosive gastritis and acute gastroduodenal ulceration as source of gastrointestinal bleeding in liver cirrhosis. *Digestion*, **7**, 129–138.

[10] Tarnawski, A. S., Sarfeh, I. J., Stachura, J., Hajduczek, A., Bui, H. X. Dabros, W. *et al.* (1988). Microvascular abnormalities of the portal hypertensive gastric mucosa. *Hepatology*, **8**, 1708–1710.

[11] Sarfeh, I. J., Tarnawski, A., Hajduczek, A., Stachura, J., Bui, H. X. Krause, J. *et al.* (1988). The portal hypertensive gastric mucosa: Histologic, ultrastructural, and functional analysis of the aspirin induced damage. *Surgery*, **104**, 79–85.

[12] Viggiano, T. R. and Gostout, C. J. (1992). Portal hypertensive intestinal vasculopathy: A review of the clinical, endoscopic, and histopathological features. *Am. J. Gastroenterol.*, **87**, 944–954.

[13] Sarin, S. K., Sreenivas, D. V. Lahoti, D. *et al.* (1992). Factors influencing development of portal hypertensive gastropathy in patients with portal hypertension. *Gastroenterology*, **102**, 994–999.

[14] Sarin, S. K., Misra, S. P., Singla, A., Throat, V. and Broor, S. L. (1988). Evaluation of the incidence and significance of the "mosaic pattern" in patients with cirrhosis, non cirrhotic portal fibrosis and extra hepatic obstruction. *Am. J. Gastroenterol.*, **83**, 1325–1339.

[15] D'Amico, G., Montalbano, L., Traina, M., Pisa, R., Menozzi, M. Spano, C. *et al.* (1990). Natural history of congestive gastropathy in cirrhosis. *Gastroenterology*, **99**, 1558–1564.

[16] McCormack, T. T., Smallwood, R., Walton, L., Martin, T., Robinson, P. and Johnson, A. (1984). Doppler ultrasound probe for assessment of bloodflow in oesophageal varices. *Lancet*, **ii**, 677–678.

[17] Grobe, J. L., Kozarek, R. A., Sanowski, R. A., Legard, J. and Kovac, A. (1984). Venography during endoscopic injection sclerotherapy of oesophageal varices. *Gastroint. Endosc.*, **30**, 6–8.

[18] Sagar, S., Grime, J. S., Little, M., Patten, M., Gulliford, P. Critchley, M. *et al.* (1983). Technitium-99 m labelled bran : A new agent for measuring gastric emptying. *Clinical Radiology*, **34**, 275–278.

[19] Kotzampassi, K., Eleftheriadis, E. and Aletras, H. (1990). The 'mosaic-like' pattern of portal hypertensive gastric mucosa after variceal eradication by sclerotherapy. *J. Gastroent. Hepatol.*, **5**, 659–663.9.
[20] Reilly, J. A., Frost, C. F., Quigley, E. M. M. and Rikkers, L. F. (1990). Gastric emptying of liquid and solids in the portal hypertensive rat. *Dig. Dis. Sci.*, **35**, 781–786.
[21] Greenholz, S. K., Hall, R. J. and Sonheimer, J. M. (1988). Consequences of oesophageal endosclerosis in children. *J. Paediat. Surg.*, **23**, 38–41.
[22] Phelps, T. O. and Mallane, J. F. (1976). Portal hypertension and gastric lesion in the rat. *Arch. Surg.*, **111**, 190–194.
[23] Bonvoisin, S., Sanzari, R., Gonnot, D., Descos, L., Francois, Y. Sappey Marinier, D. *et al.* (1992). Etude scintigraphique de la vidange gastrique apres sclerotherapie endoscopique de varices oesophagiennes chez les malades atteints de cirrhose alcoholique. *Gastroenterol. Clin. Biol.*, **16**, 812–821.
[24] Stein, R. and Scott Brooks, W.Jr. (1985). Delayed gastric emptying following endoscopic sclerosis of oesophageal varices. *Gastroenterology*, **88**, A 1598.
[25] Clarke, R. J. and Alexander Williams, J. (1973). The effect of preserving antral innervation and of a pyloroplasty on gastric emptying after vagotomy. *Arch. Surg.*, **102**, 43–44.
[26] Glysteen, J. J., Burdeshaw, J. A. and Hallenbeck, G. A. (1976). Gastric emptying of liquids after different vagotomies and pyloroplasty. *Surg. Gynaecol. Obstet.*, **142**, 41–48.
[27] Nilsson, G., Yallow, R. S. and Berson, S. A. (1973). Distribution of gastrin in the gastrointestinal tract of human, dog, cat and hog. In : *Frontiers in Gastrointestinal Hormone Research.* Eds. Anderson, Almquist and Wiksell, Stockholm, pp. 95–101.
[28] Thuluvath, P. J. and Triger, D. R. (1989). Autonomic neuropathy and chronic liver disease. *Quart. J. Med.*, New Series 72, **268**, 737–747.
[29] Khosla, S. N., Saynal, S. and Nand, N. (1991). Autonomic functon tests and clinical significance of dysautonomia in chronic liver disease. *J. Asso. Phys. Ind.*, **39**, (12), pp. 924–926.
[30] Isobe, H., Sakai, H., Satoh, M., Sakamoto, S. and Nawata, H. (1994). Delayed gastric emptying in patients with liver cirrhosis. *Dig. Dis. Sci.*, **39**(5), 983–987.

### COMMENTARY

Though it has been appreciated for some time that gastrointestinal symptoms are common in patients with chronic liver disease, until recently few studies had documented the true frequency of any specific gastrointestinal symptoms are common in patients with chronic liver disease, until recently few studies had documented the true frequency of any specific gastrointestinal symptom or symptom complex in liver disease [31]. Furthermore, little is known of the pathophysiology of these symptoms and their relationship to gut function. Over the past several years, a number of investigator have attempted to evaluate some aspects of gastrointestinal function in patients with portal hypertension and chronic liver disease. Particular attention has been focused on gastric motor dysfunction.

Stimulated, perhaps, by the description of gastric mucosal changes related to portal hypertension [32] and of related effects on acid secretion and gastric mucosal defense [33], a number of reports have documented alterations in gastric emptying in patients with liver disease and portal hypertension [33–36]. The precise nature of this effect, however, remains controversial, with some studies demonstrating an acceleration and others a delay in gastric emptying. Many factors, including inter-species differences, the severity of underlying liver disease and the relative predominance of effects on regional gastric function might explain these discrepancies. Thus, an effect on the gastric fundus could result in an impairment in receptive relaxation and accelerated emptying, while altered antral contractibility would delay emptying. One could also speculate that reductions in fundic compliance could lead, through the generation of high post-prandial intragastric pressures, to the precipitation of transient lower esophageal sphincter relaxation, and, thereby, gastroesophageal reflux [37].

In this issue of the journal, Balan and his colleagues address another variable which might influence gastric emptying, in patients with portal hypertension who are treated with endoscopic sclerotherapy. Their provocative hypothesis suggests that sclerotherapy might result in a chemical vagotomy, and thereby exert typical vagotomy effects on liquid and solid emptying from the stomach.

Esophageal dysfunction following injection sclerotherapy is, indeed, an important clinical phenomenon [38]. For the most part, however, esophageal dysfunction following sclerotherapy is related to mucosal ulceration and stricture formation. Sclerotherapy has also been associated with changes in esophageal motor function, which include impaired peristaltic amplitude and an increased incidence of abnormal contractile waves in the lower esophagus [39]. This study, however, is the first to examine sclerotherapy effects on the stomach. While failing to observe any difference in gastric emptying rate between their portal hypertensive patients as an entire group and healthy volunteers, Balan and colleagues report that patients who received injection sclerotherapy demonstrated more rapid emptying of a semi-solid meal than those who had not. Furthermore, emptying rate appeared to correlate with both the number of sclerotherapy injections and the total volume of sclerosant injected.

These findings suggest that prior sclerotherapy may be yet another variable which could affect gastric emptying in patients with portal hypertension and liver disease, and contribute to the variability that has been noted among various studies. While interpretation of their study is limited by a number of factors including a relative paucity of information on control subjects, a failure to separately evaluate solid and liquid emptying and a lack of correlation with symptoms or time from sclerotherapy, this study suggests, at the very least, that a history of prior sclerotherapy should be factored into an evaluation of gastrointestinal motor function in this patient group. Based on prior experience, one would expect vagotomy to result in an acceleration of liquid emptying and a retardation of solid emptying; why emptying of a semi-solid meal should have been accelerated in this study remains unclear. Further direct studies of vagal function are required and will need to factor in the known prevalence of autonomic dysfunction in patients with chronic liver disease, *per se* [40].

Given the high prevalence of gastrointestinal symptoms suggestive of gastric motor dysfunction among patients with liver disease and portal hypertension, further prospective studies of gastric function in this patient group are warranted, in doing so, one must obviously take into account the many factors which may contribute to gastric motor dysfunction in this population [41].

Eamonn M. M. Quigley MD, FRCP
Layton F. Rikkers MD
Departments of Internal Medicine and Surgery
University of Nebraska Medical Center
600 So 42nd St.
Omaha, Nebraska 68198-2000

### *References*

[31] Galati, J. S., Monsour, H. P., Dyer, C. H., Seagren, S. and Quigley, E. M. M. (1995). A survey of the frequency of gastrointestinal complaints in patients with chronic liver disease. *Gastroenterology*, **108**, A 1068.
[32] Viggiano, T. R. and Gostout, C. J. (1992). Portal hypertensive intestinal vaculopathy: a review of the clinical, endoscopic, an histopathological features. *Am. J. Gastroenterol.*, **87**, 944–954.
[33] Sarfeh, I. J., Tarnawaski, A., Maeda, R., Raymont, K., Mason, G. R. and Ivey, K. J. (1984). The gastric mucosa in portal hypertension: Effects of topical bile acid. *Scand. J. Gastroenterol.*, **92**, (Suppl): 89–194.
[34] Reilly, J. A., Forst, C. F., Quigley, E. M. M. and Rikkers, L. F. (1990). Gastric emptying of liquids and solids in the portal hypertensive rat. *Dig. Dis. Sci.*, **35**, 781–786.
[35] Isobe, H., Sakai, H., Satoh, M., Sakamoto, S. and Nawata, H. (1994). Delayed gastric emptying in patients with liver cirrhosis. *Dig. Dis. Sci.*, **39**, 983–987.
[36] Galati, J. S., Holdeman, K. P., Dalrymple, G. V., Harrison, K. A. and Quigley, E. M. M. (1994). Delayed gastric emptying of both the liquid and solid components of a meal in chronic liver disease. *Am. J. Gastroenterol.*, **89**, 708–711.
[37] Quigley, E. M. M. (1995). Gastrointestinal dysfunction in liver disease. *Rom. J. Gastroenterol.*, **4**, 131–132.
[38] Quigley, E. M. M. and Zetterman, R. K. (1992). Gastrointestinal complications of chronic liver disease. In: *Complications of chronic liver disease.* Rector W. G. Jr, Ed. Chicago, Mosby Year Book, pp. 317–338.
[39] Grande, L., Planas, R., Lacima, G., Boix, J., Ros, E., Esteve, M., Morillas, R. and Gasull, M. A. (1991). Sequential esophageal motility studies after endoscopic injection sclerotherapy: a prospective evaluation. *Am. J. Gastroenterol.*, **86**, 36–40.
[40] Hendrickse, M. T., Thululath, P. J. and Triger, D. R. (1992). Natural history of autonomic neuropathy in chronic liver disease. *Lancet.*, **399**, 1462–1464.
[41] Quigley, E. M. M. (1996). Gastrointestinal dysfunction in liver disease. *Dig. Dis. Sci.*, (In Press).

The precise mechanisms responsible for the development of portal hypertensive gastropathy (PHG) are unknown.

The aim of this study was to examine the role of injection sclerotherapy of oesophageal varices in the development of PHG.

Gastric emptying was studied using a radio nuclide test meal with the emptying characteristics of a slow liquid in 57 patients with cirrhosis (of whom 34 had received injection sclerotherapy for their oesophageal varices) and 20 normal healthy volunteers.

The speed of gastric emptying is correlated directly with the number of injections and the volume of sclerosant injected.

The injection sclerotherapy has been reported to exacerbate the PHG (5–14–15):

- one possible explanation is the getting worse of the gastric mucosal congestion after the obliteration of lower oesophageal veins.
- a further possibility is the extravasation of sclerosant into the mediastinum with a vagal damage and a delay in the rate of gastric emptying.
- Moreover the extension of the exposure of gastric mucosa to the acid and pepsin may exacerbate PHG.

A very large number of patients with oesophageal varices would have to be followed to elucidate the effects of injection sclerotherapy, on vagal function.

A more precise evaluation of "Chemical vagotomy" requires ph-metry and manometry.

I hope that other studies bring into the rate of gastric emptying before and after injection sclerotherapy with a multicentre controlled trial.

Prof. D. F. D'Amico
Prof. Malcom Puntis
HPB Editorial Office
University Department of Surgery
University of Wales; College of Medicine
Heath Park, Cardiff CF4 4XN, UK

